# Host tissue stoichiometry links nutrient availability to parasite abundance in a piscivorous fish

**DOI:** 10.1007/s00442-026-05971-w

**Published:** 2026-09-04

**Authors:** Adrienne Stanley, Isabella Sadler, Scott Binger, Charlotte F. Narr

**Affiliations:** School of Biological Sciences, Southern Illinois University in Carbondale, Carbondale, IL USA

**Keywords:** Largemouth Bass, Nitrogen, Phosphorous, Trematode, Trophic interactions

## Abstract

Understanding how ecosystem nutrient availability influences parasites is challenging, especially for trophically transmitted parasites in hosts occupying higher trophic levels. Host tissue nutrient stoichiometry may elucidate these effects by indicating nutrient availability within a host’s environment and/or mediating these effects on parasites. We assessed these possibilities by manipulating nutrient availability in 11 experimental ponds stocked with piscivorous largemouth bass for 3 months and quantifying the role of a digestive tissue’s stoichiometry in mediating the relationship between basal nutrient availability, the abundance of both fish and invertebrate consumers (including potential intermediate hosts), and the abundance of the trematode parasite, *Crepidistomum* spp., in this tissue. Fertilization increased the total phosphorus (P) and decreased the carbon (C):P and nitrogen (N):P ratios in the water column and fine particulate organic matter of ponds. While we did not detect effects of fertilization on intermediate trophic levels, N:P ratios in the bass digestive tissue were inversely related to both total water column P levels and the abundance of *Crepidistomum* spp. in these tissues. Overall, the nutrient ratios of a key tissue involved in predator digestion mirrored fertilization-induced shifts in basal nutrient availability and explained more variation in the abundance of a trophically transmitted parasite than any other variable we quantified. Whether tissue nutrient ratios directly influenced the abundance of this parasite or simply reflected shifts in the ecosystem that mediated parasite abundance remains unclear. Regardless, digestive tissue nutrient stoichiometry appears to provide an informative link between basal nutrient availability and parasite abundance in these tissues.

**Supplementary Information:**

The online version contains supplementary material available at https://doi.org/10.1007/s00442-026-05971-w.

## Introduction

The enrichment of freshwater ecosystems with limiting nutrients, nitrogen (N) and phosphorus (P), has been accelerated in recent decades by anthropogenic forces (Gilbert & Buford 2017). Increases in parasitism associated with freshwater nutrient loading have been documented for decades (Hartmann and Nümann 1977; Hirshfield et al. 1983; Okamura, 2011). However, discerning the mechanisms driving these trends is difficult given the multitude of factors affected by nutrient loading (Lafferty 1997; Budria 2017), the diversity of parasites (Dobson et al. 2008), and complex parasite life cycles involving multiple hosts.

While host and parasite abundances are often correlated (Arneberg et al. 1998), the net effect of nutrient enrichment on parasites that require multiple hosts to complete their life cycle (i.e. trophically transmitted parasites) is challenging to quantify and predict. Elevated nutrient availability can increase the quality and quantity of primary producers, leading to increases in primary consumers (La Pierre and Smith 2016). When these consumers serve as first intermediate hosts, this can translate into increased abundances of parasites in secondary hosts (Johnson et al. 2007). However, nutrient additions can also alter the community composition of primary producers and consumers (Fairchild et al. 1985; Stelzer and Lamberti 2001; Nessel et al. 2021), which could alter the abundance of intermediate hosts in unexpected ways. In freshwater ecosystems, nutrient-induced increases in primary production can also create hypoxic conditions (Watson et al. 2016) that reduce the abundance of free-living (host) species and negatively impact parasite abundance (Rao et al. 2014). Conversely, nutrient-induced hypoxia has also been suspected of increasing the abundance of parasites in a definitive host by altering spatial overlap between first and second intermediate hosts (Marcogliese et al. 1990). Overall, the natural complexity of these systems and the observational nature of much of the work examining relationships between basal nutrients and the abundance of trophically transmitted parasites in definitive hosts limits our understanding of nutrient-mediated shifts in the abundance of parasites within definitive hosts.

In addition to shifting host abundances, nutrients at the base of the food web may also influence parasites via shifts in the diet quality of individual hosts (Frost et al. 2008). However, for definitive hosts occupying upper trophic levels (e.g. predators), quantifying the effect of basal nutrients through multiple lower trophic levels up to the diet quality of the host is challenging. To link ecosystem nutrient availability to shifts in consumer diet quality across multiple trophic levels, it is useful to adopt a framework that converts nutrients to a common currency. Ecological stoichiometry uses elements such as carbon (C), N, and P and the law of conservation of mass to observe nutrient transfer through natural systems (Sterner and Elser 2002). This framework considers the ratios of these essential nutrients in ecosystems and individuals, which can be used to compare how well-matched organisms are to their environments and food sources (Persson et al. 2010). Thus, quantifying the relationship between basal nutrient ratios, the abundance of animals in intermediate trophic levels, and nutrient ratios in definitive hosts occupying upper trophic levels could improve our understanding of nutrient effects on the diet quality of definitive hosts.

Ecological stoichiometry could also be a powerful predictor of host-parasite interactions (Aalto et al. 2015; Bernot and Poulin 2018; Sanders and Taylor, 2018). For example, it may be possible to anticipate which parasites will be most successful in a host by comparing the nutrient composition of specific parasites to those of their host (Frenken et al. 2021). Observational studies applying this framework to macroparasites in vertebrates have shown that the nutrient composition of parasites are distinct from those of their host (Grunberg et al. 2024) and are linked to characteristics of the parasite (e.g. size and life cycle stage; Paseka and Grunberg 2019). However, clear predictions of winners and losers within parasitic communities remain elusive. This lack of clarity may be, in part, because the nutrient composition of an animal host varies across tissues (Sterner and Elser 2002), and parasites may be more tightly linked to the nutrient composition of the specific tissues (i.e. infection site) they inhabit than the nutrient composition of the entire host. Within some infection sites, especially those directly involved in food absorption, this nutrient composition also exhibits pronounced variation across individual hosts, potentially due to variation in host diet (Richardson and Nickol 1999; Stanley et al. 2024). Consistent with predictions from ecological stoichiometry, this variation in site nutrient stoichiometry across individual hosts explained variation in the abundance of parasites occupying it (Stanley et al. 2024). However, this observational study did not placed these relationships within the nutritional environment of the host. As a result, it is unclear if the abundance of nutrients at the base of the food web can influence the nutrient composition of specific infection sites in definitive hosts in ways that are linked to the abundance of parasites at these sites.

**Fig. 1:**
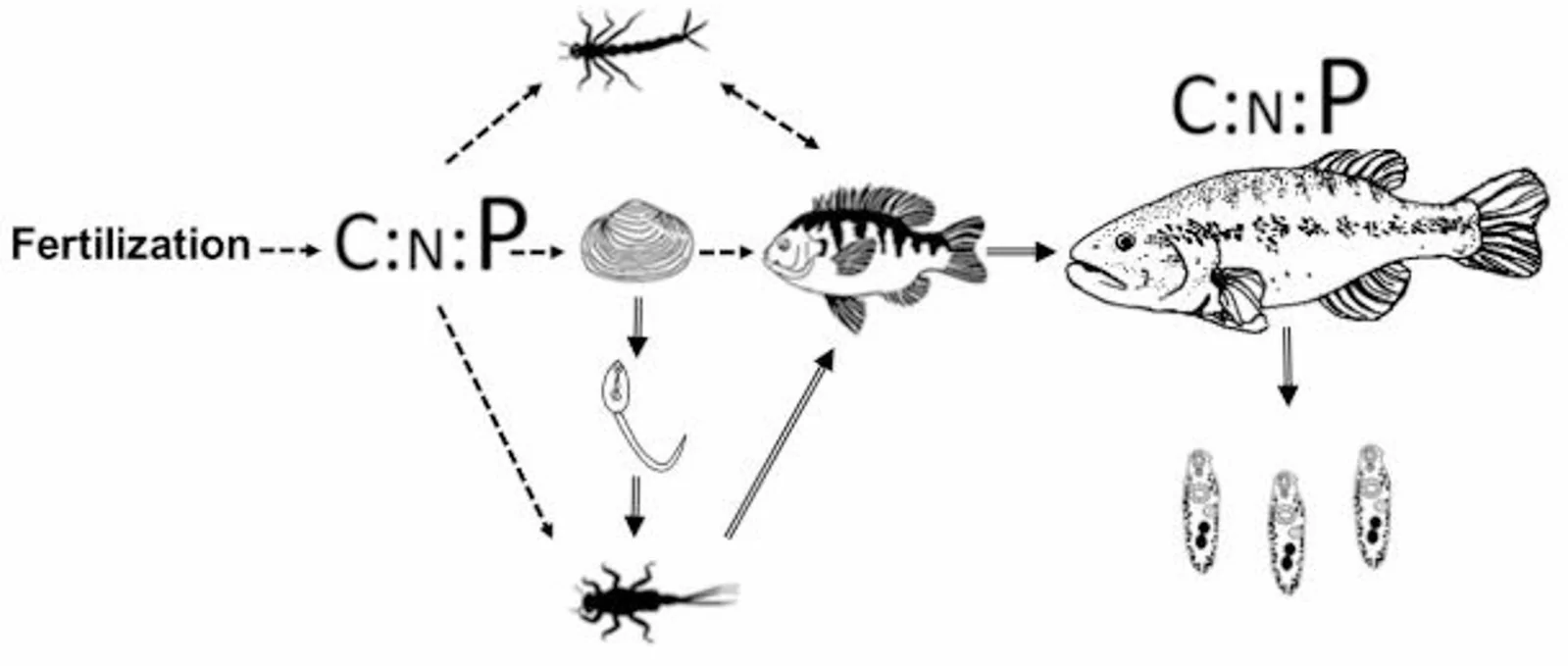
Overview depicting the primary pathways through which we expected fertilization to alter the abundance of *Crepidostomum spp*. in largemouth bass in our experimental ponds system. For simplicity, only single, direct effects from one component to another are shown. The transmission route of the parasite (from 1st intermediate host through the definitive host) is outlined in arrows with double lines. *Crepidostomum spp*. cercaria are released from the first intermediate host (a sphaerid clam) and infect a second intermediate host (shown here as a mayfly). A paratenic host (shown here as a bluegill) may eat the second intermediate host and facilitate its transmission, through ingestion, to the definitive host (here, a largemouth bass) where it matures into an adult in the pyloric caecum (not shown). Note that ingestion of the second intermediate host can also produce infections in largemouth bass, but the fish used in our experiment were large enough to have made the switch to a piscivorous diet. We expected that fertilization would influence nutrient availability at the base of the food web by increasing total phosphorus (P) availability relative carbon (C) and nitrogen (N) in the water column and fine particulate organic matter. These shifts could increase the abundance of intermediate or paratenic hosts by increasing the quality and quantity of their diet (for the paratenic host, this diet includes other, non-host invertebrates, shown here as a dragonfly). These bottom-up effects could alter largemouth bass nutrient content, especially in the pyloric caeca, and facilitate the development and transmission of the parasite. Drawings by Adrienne Stanley

Here, we investigate how the abundance of a trophically transmitted parasite is linked to shifts in basal nutrient availability via shifts in the stoichiometry of its infection site within its definitive host. Specifically, we examined how nutrient additions influence the abundance of the trematode parasite *Crepidostomum* spp. in the pyloric caecum, a digestive tissue, of largemouth bass (*Micropterus salmanoides*) in experimental ponds with well-developed communities (Fig. 1). Trematodes within the genus *Crepidostomum* typically utilize sphaerid clams as first intermediate hosts and may use a variety of insects or crustaceans as second intermediate hosts (Hoffman and Williams 1999). Planktivorous fish can also become infected by *Crepidostomum* spp. and play an important role as paratenic hosts (hosts that are not crucial to parasite development but facilitate transmission into higher trophic level definitive hosts, Médoc et al. 2011). We focused on the pyloric caecum because, in addition to being the primary infection site of *Crepidostomum* in bass, it is an important site of nutrient absorption in largemouth bass, and thus is likely to reflect changes in food quality or quantity (Buddington and Diamond 1986). For example, in green sunfish, the pyloric caecum has elevated total protein, free amino acids, and aminopeptidase activity for up to 16 to 32 h (in the case of total protein) after feeding (Richardson and Nickol 1999). We quantified the effects of fertilization on the total nutrient content of the water column and the nutrient ratios of primary producers (fine particulate organic matter). Then, we examined links between the nutrient ratios of these basal resources, the abundances of invertebrates and fish prey for largemouth bass, the nutrient stoichiometry of bass pyloric caeca, and the abundance of a trematode parasite, *Crepidostomum* spp., in this organ.

## Materials and methods

### Study Site and experimental design

We conducted our experiment in twelve 0.04-hectare ponds located at the outdoor experimental pond facility at the Touch of Nature Environmental Center outside of Carbondale, IL. The ponds used in this study were approximately 1–2 m deep, had been filled with water for at least several months prior to our experiment, and had existing fish communities composed of primarily planktivorous species (Online Resource 1, Table 1). After the completion of an initial round of sampling for invertebrates (mid May-late June) and fish (June 22nd through July 2nd ), fertilization was initiated in half of the ponds on July 9th and continued through the end of September. Bass were added to all ponds approximately one week after fertilization commenced. Invertebrates were resampled between early September and mid October, and fish communities were resampled and bass were retrieved between September 25th and October 10th. While we do not have previous data on the seasonality of the *Crepidostomum spp.* life cycle at our study location, our study was conducted at a time when active transmission between each host in the lifecycle was likely. There is some evidence that *Crepidostomum spp.* prevalence exhibits little seasonality in largemouth bass at locations near our study site (i.e. Northern Missouri; Banks and Ashley 2000), but the abundance of *C. cronutum* in bluegill peaked during August in a lake in Arkansas (Cloutman 1975). Work at locations further north of our study site (in Michigan) indicate that *C. cooperi* is actively infectious in two secondary intermediate hosts (mayflies and amphipods) from June through at least August and then tapers off in cooler months (Hazen and Esch 1977; Esch and Hazen 1982). We sampled each pond for total water column nutrients and dissolved oxygen twice in the two weeks before fertilization began and approximately every one to two weeks throughout the fertilization period (Online Resource 1, Fig. 1). During each sampling period, we interspersed our sampling of the ponds from each treatment temporally so that one haphazardly selected pond was sampled from each treatment before we sampled another replicate pond from the same treatment. To minimize the likelihood of preexisting differences in pond nutrient ratios confounding the effects of fertilization, we measured the total water column nutrient ratios in ponds prior to assigning them a treatment. Then we randomly assigned ponds to fertilized or control (unfertilized) treatments, until we achieved similar starting conditions across treatments.

**Table 1:** Contrasts between 6 fertilized and 6 unfertilized experimental ponds before and toward the end of (After) a 3 month fertilization treatment for water column mean and minimum (min) dissolved oxygen (DO), total carbon (TC), nitrogen (TN), phosphorus (TP), and natural log-transformed molar ratios of these nutrients (ln TC: TN, ln TC: TP, and ln TN: TP). Contrasts represent differences between estimated marginal means obtained from linear mixed models including the fertilization treatment, time (before and after), and a random effect of pond. Estimates indicate the difference between fertilized and unfertilized ponds within each time period with positive values indicating higher values in fertilized ponds and negative values indicating lower values in fertilized ponds. Significant differences (α = 0.05) between treatments are in bold. Estimate, standard error (SE), degrees of freedom (*d.f.*), t- ratios (*t*), and p values (*p*) are shown

Measurement	Time	Estimate	SE	d.f.	t	*p*
**Mean DO (mg/L)**	**After**	**-4.38**	**1.30**	**16.7**	**-3.37**	**0.0037**
	Before	-0.82	1.49	29.3	-0.55	0.58
**Min DO (mg/L)**	**After**	**-1.95**	**0.51**	**17.8**	**-3.84**	**0.0012**
	Before	-0.686	0.74	33.3	-0.93	0.36
**TC (mg/L)**	**After**	**1.47**	**0.568**	**103**	**2.59**	**0.011**
	Before	1.92	1.06	103	1.80	0.075
TN (mg/L)	After	-0.072	0.049	103	1.47	0.15
	Before	-0.047	0.092	103	0.508	0.61
**TP (mg/L)**	**After**	**0.066**	**0.013**	**103**	**5.19**	**< 0.0001**
	Before	-0.018	0.24	103	0.74	0.46
**ln TC: TN**	**After**	**0.17**	**0.057**	**103**	**2.917**	**0.0043**
	Before	0.175	0.11	103	1.64	0.10
**ln TC: TP**	**After**	**-0.39**	**0.11**	**103**	**-3.67**	**0.0004**
	Before	0.29	0.2	103	1.43	0.16
**ln TN: TP**	**After**	**-0.52**	**0.10**	**103**	**5.09**	**< 0.0001**
	Before	0.096	0.19	103	0.51	0.61

Every two weeks, treatment ponds were sprayed with 0.1 L of liquid ammonium polyphosphate (N: P_2_O_5_) fertilizer (10-34-0). This amount was chosen to reflect the 100 pounds of fertilizer per surface acre recommended by the Illinois Department of Natural Resources (2017) to increase productivity in new fishponds. We stocked 7 bass, ranging in length from 112 mm to 344 mm, and weight from 69 g to 438 g, into each pond (Fertilized ponds: average length = 222 mm, SD = 24 mm; average mass = 163 g, SD = 27 g; Unfertilized ponds: average length = 221 mm, SD = 30 mm; average mass = 175 g, SD = 27 g). These fish were collected using a mix of seining and rod and reel capture from several other ponds on site, where their diet had been supplemented with pellet feed (SIU IACUC # 21 − 019). All bass stocked into the ponds were large enough to have made the switch to a primarily piscivorous diet. Although the considerable range of sizes and variability in the types of prey between ponds likely impacted the diet of the bass, based on previous studies of largemouth bass (Timmons et al., 1980) we included the following fish as potential prey items for our bass: small largemouth bass, Crappie of age class 2 and under (Jackson and Hurley 2005), all Green Sunfish (Savitz and Janssen 1982), all Golden Shiners (Christensen and Moore 2007), Bluegill of age class 3 and under (Garling and Ashley 2002).

## Sampling

We measured dissolved oxygen (DO) and total C (TC), N (TN), and P (TP) every 6 or 7 days from June 28th through August 17th and then again on September 5th and 21st (roughly 18 days apart). Samples for total water column nutrients were filtered through a 60 μm mesh in the field. DO was measured continuously in each pond for approximately 48 h during each time period using deployable dissolved oxygen loggers (PME miniDOT). Approximately two to four weeks prior to the addition and retrieval of bass hosts to and from ponds, we sampled benthic invertebrates and zooplankton in each pond. Fine particulate organic matter (FPOM) was only sampled prior to the retrieval of bass and was accomplished by collecting 1 L of water from each pond and filtering it onto a 0.7 μm pore size glass fiber filter. Invertebrates were sampled using benthic net sweeps done at three depths (0.5 m, 1 m, and maximum wadable depth) for three rounds in each pond. Each round lasted 1 min along a 2 m length of pond. Zooplankton samples were taken from the same depths using a vertical tube sampler (Graves and Morrow 1988). Samples from each round and depth were poured through a 1 mm mesh sieve and the material remaining on the sieve from all three depths and rounds were combined. To measure fish species assemblages and recapture the largemouth bass in the ponds, we seined the full length of the ponds using a 6ft deep seine with 1/8in mesh. Fish were counted, measured by length, grouped into a general age class, and the presence of tadpoles was noted. Largemouth bass caught at the end of the fertilization period were euthanized by immersion in Tricaine methanesulfonate (MS 222) at concentrations of 300 mgL^−1^for at least 30 min following American Veterinary Medical Association guidelines (Underwood and Anthony 2020) and then frozen. All other animals caught while seining were returned to the pond they came from.

## Processing of samples

Invertebrates were sorted and identified to family using Merritt and colleagues (2005). We thawed and dissected a total of 34 largemouth bass (two to four haphazardly selected individuals from each pond) and removed their pyloric caeca (Hoffman and Williams 1999). Each pyloric caecum was scraped then washed, and the diluent was allowed to settle in a conical container. The fluid was then decanted by pouring out the upper two thirds of the solution. If the remaining diluent was murky, more distilled water was added and the contents were allowed to resettle. This process was repeated until the diluent was clear, and the remaining matter was searched for parasites. Each pyloric caecum was viewed under a dissecting scope and searched visually for *Crepidostomum* spp. Then, a small subset of this tissue was separated and picked clean of connective tissue and fat. These subsets were dried at 50 °C to a constant weight. Dried samples of FPOM and fish pyloric caeca were weighed, homogenized, and subsampled for total C, N, and P. We analyzed tissue P concentrations using the ascorbic acid molybdenum-blue method after digestion in potassium persulfate (APHA 1992). C and N were analyzed on an elemental analyzer (Thermo Scientific Flash 2000).

## Statistical analyses

### Effects of fertilization on water chemistry and primary producers

Statistics were conducted in R (version 4.3.0, R Core Team 2023) and R studio (Posit Team 2024). All ratios are molar and were natural log-transformed (ln-transformed) to avoid biases in interpretation (Isles 2020). To assess the effects of fertilization on DO and the total nutrient content and ratios of the water column, we used linear mixed models (R package lme4, Bates et al. 2015) to compare minimum and mean DO, and aquatic TC, TN, TP, C:N, C:P, and N: P in fertilized and unfertilized ponds before and after we began fertilizing. Each model included fixed effects of fertilization, a binary time variable (before or after fertilization, which included all sampling dates before or after fertilization began), an interaction between these two variables, and pond as a random factor (intercept). We used estimated marginal means (R package emmeans, Lenth 2024) to determine if the nutrient content and ratios of ponds in the fertilized treatment differed from those of ponds in the unfertilized treatment before and after fertilization began. Because we did not have data on FPOM nutrient concentrations before our treatment, we assessed the effect of fertilization on FPOM nutrient concentration (µgL^− 1^ of C, N, P) or nutrient ratios (C: N, C: P, and N: P) using Welch’s two sample t-tests.

### Effects of fertilization on intermediate hosts and the consumer community

We conducted model selection using Akaike’s information criterion (AICc, R package AICcmodavg, Mazerolle 2023) to determine which variables best explained the abundances of natural log (ln)-transformed benthic invertebrates, ln-transformed zooplankton, rank-transformed sphaerid clams, and rank-transformed fish prey. Each response variable included a candidate model set comprised of univariate linear regressions (given sample size limitations in pond replication). These model sets captured: (1) the experimental treatment; (2) post-manipulation nutrient levels; (3) primary producers, (4) bottom-up/top-down forces; and (5) the pre-experiment state of the pond. For experimental treatment, we included a single candidate model with fertilization status as the predictor variable. For post-manipulation nutrient levels, we included eight candidate models, each containing one of the water chemistry parameters (minimum DO, mean DO, TC, TN, TP, C:N, C:P, and N: P). For primary producers, we ran six candidate models containing variables for FPOM nutrient concentrations (FPOM C, FPOM N, FPOM P) and FPOM nutrient ratios (FPOM C: N, FPOM C: P, and FPOM N: P). When invertebrate taxa were the response variable (i.e., zooplankton or benthic invertebrates), we included a single candidate model for fish abundance (rank-transformed) to account for the possibility of top-down effects. When fish prey were the response variable, we included two candidate models for zooplankton abundance and benthic invertebrate abundance (ln-transformed) to account for bottom-up effects. For the pre-experiment state of the pond, we included a single candidate model containing the pre-treatment abundance of the response group. Finally, each model set included an intercept-only null model. The inclusion of these univariate models resulted in eighteen candidate models per response variable (nineteen for models predicting fish prey). Models with a delta AICc < 2 were considered competitive top models.

## Variation in bass quality (pyloric caeca nutrient composition and bass length) across ponds

A similar AICc framework was used to explore the factors that shape seven response variables indicative of host quality: bass length, pyloric caecum nutrient content (%C, %N, %P) and nutrient ratios (C: N, C: P, N: P). These model sets included all three bottom-up effects: zooplankton, benthic invertebrates, and fish prey. Because all predictors were at the pond scale, we analyzed the effects on average fish length and pyloric caecum nutrient content. Running these analyses at the pond (rather than individual fish) scale avoided the need for a random effect of pond that would have competed with our fixed effects in describing variation across ponds (the primary goal of the analysis).

## Variation in Crepidostomum spp. abundance across individual hosts

The abundance of *Crepidostomum spp.* in the pyloric caeca of individual fish was modeled using hurdle models (R package glmmTMB, Brooks et al. 2017). For this analysis, individual fish were used as the unit of replication because we were interested in the role of both pond-level and individual fish-level predictors of parasite abundance. The zero-count process was modeled with the intercept only. The positive count process was modeled using a truncated poisson distribution that included a random effect of pond to account for the non-independence of fish that occupied the same pond during the experiment. We also included the primary source pond for fish in each experimental pond as an additional random effect to account for potential effects of the fish’s original rearing pond on final *Crepidostomum* spp. abundance. For the positive count process, we again, wanted to capture the potential indirect effects of variation across ponds in terms of (1) the experimental treatment, (2) post-manipulation nutrient levels, (3) primary producers, (4) primary (benthic invertebrates, zooplankton) and secondary (fish prey) consumers, and (5) first intermediate hosts (sphaerid clams). In addition to these pond-level predictors, our list of candidate models explaining variation in *Crepidostomum spp.* was expanded to include individual host-level characteristics including bass length or the nutrient contents or nutrient ratios of the pyloric caecum of each fish. Because our sample size was greater for *Crepidostomum spp.*, we considered every possible combination of a single predictor at the host scale and a single predictor at the pond scale. This yielded a set of 90 candidate models. For simplicity we report only the top 20 models as indicated by model selection. We checked for overdispersion using the R package “performance”. We removed one fish from this analysis because it had strong leverage (as indicated by a cook’s distance > 5x the mean cook’s distance).

## Results

### Effects of fertilization on water chemistry and primary producers

There were no differences in mean or minimum DO or water column nutrients between fertilized and unfertilized ponds in the two sampling dates prior to fertilization (Table 1). During our fertilization period, fertilized ponds had elevated water column TC, TP, and TC: TN and reduced mean and minimum DO, TC: TP and TN: TP compared to unfertilized ponds (Table 1). Two to four weeks before fertilization ended, fertilized ponds had lower suspended FPOM C: P (t_7.5_=3.41, *p* = 0.010) and N: P (t_6.2_=4.74, *p* = 0.0029) than unfertilized ponds. We did not detect effects of fertilization on FPOM C: N (t_9.4_=-0.14, *p* = 0.89).

### Effects of fertilization on intermediate hosts and the consumer community

**Fig. 2:**
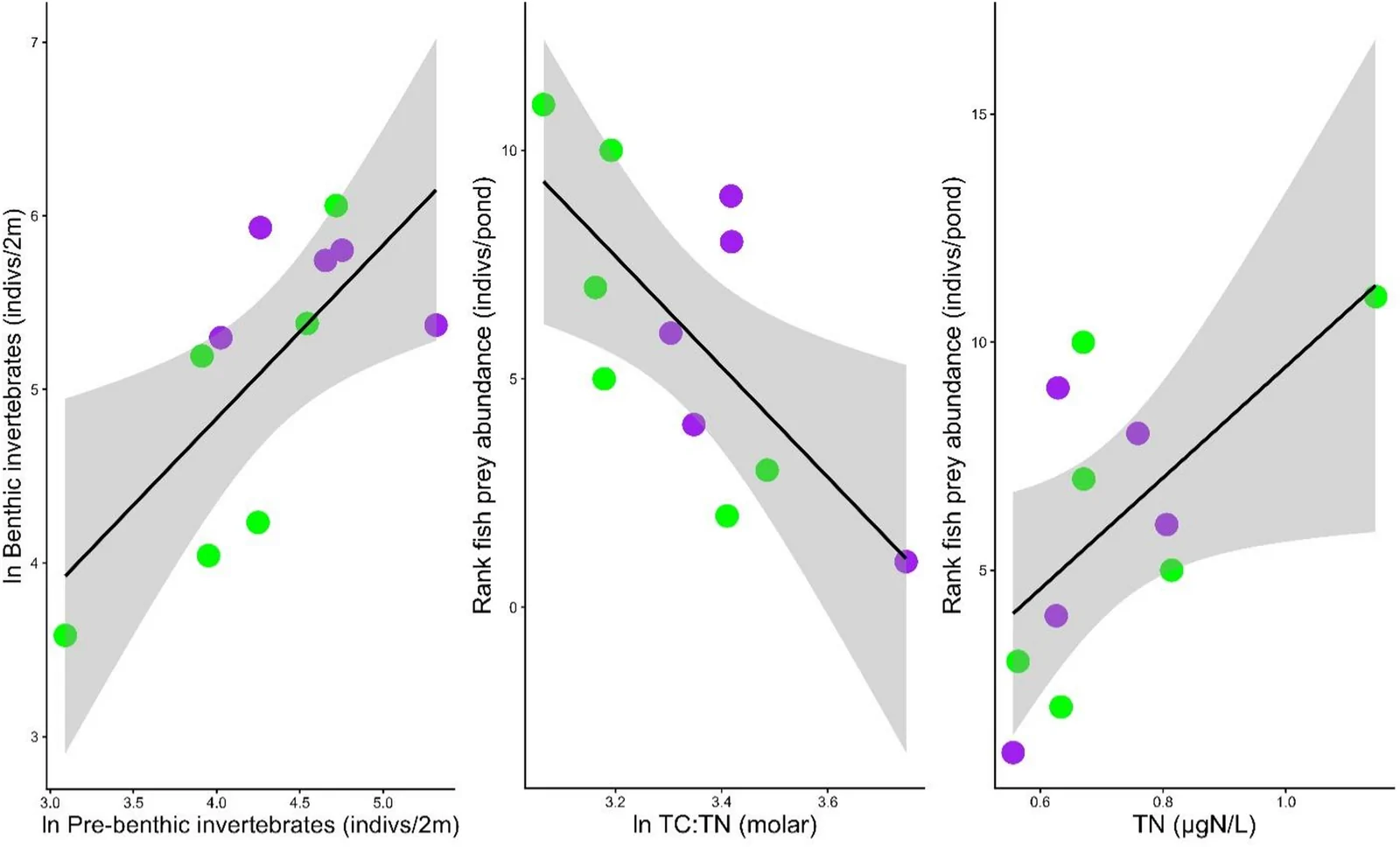
Relationship between natural log-transformed (ln) benthic invertebrate abundances (individuals/sample) before and after fertilization and rank transformed fish abundances and water column total carbon (TC): total N (TN) ratios, and TN, across fertilized (*N* = 5, purple) and unfertilized (*N* = 6, green) ponds. Shaded regions indicate standard error

Benthic invertebrate abundances at the end of the experiment were best predicted by their abundance before fertilization began (Online Resource 1, Table 2, β = 1.00, SE = 0.335, t_9_ = 2.98, *p* = 0.016, R^2^ = 0.50, Fig. 2). The null model was among the top models predicting zooplankton abundance, sphaerid clam abundance, and final bass length (Online Resource 1, Table 2). The top models for possible fish prey abundance (Online Resource 1, Table 2) showed that fish prey abundance was inversely related to water column TC: TN ratios (β =-12.10, SE = 4.20, t_9_=-2.88, *p* = 0.018, R^2^ = 0.48, Fig. 2) and positively related to TN (β = 12.20, SE = 5.21, t_9_ = 2.34, *p* = 0.044, R^2^ = 0.38, Fig. 2).

### Variation in bass quality (pyloric caeca nutrient composition and bass length) across ponds

**Fig. 3:**
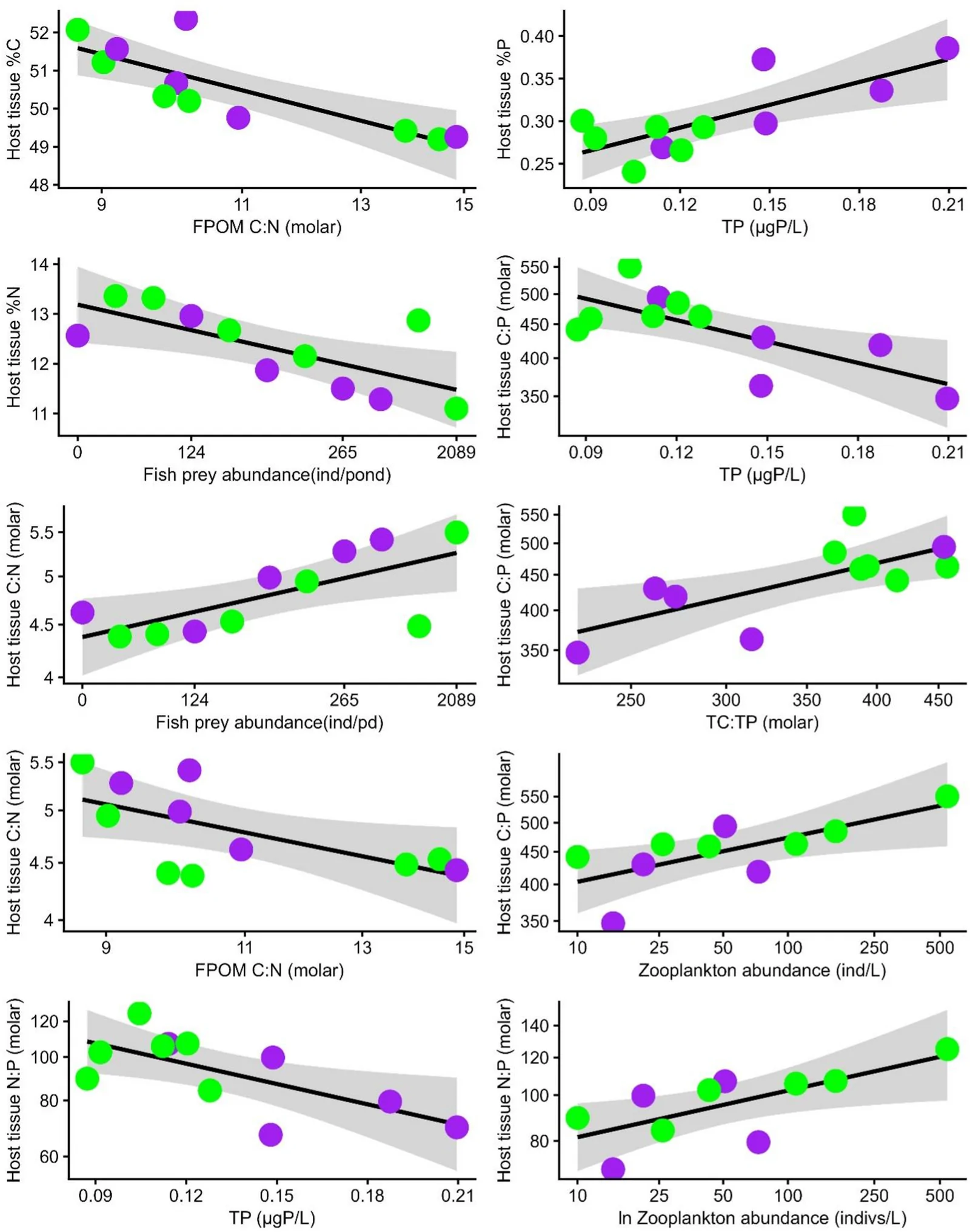
Relationship between average pyloric caeca % carbon (C), nitrogen (N), and phosphorus (P), C:N, C:P and N:P ratios of largemouth bass in fertilized (*N* = 5, purple) and unfertilized (*N* = 6, green) and natural log (ln)-transformed fine particulate organic matter (FPOM) C:N ratios, rank (rnk) transformed fish prey abundances, natural log transformed (ln) zooplankton abundances, and water column total (T) P and TC:TP ratios. Note that the axes are labeled with untransformed values, but the axes for nutrient ratios and rank transformed variables have been transformed. Shaded regions indicate standard error

Top models for pyloric caeca tissue nutrient composition (Online Resource 1, Table 3) showed that the average %C and C: N ratio of bass pyloric caeca were negatively related to pond FPOM C: N ratios (host tissue %C: β =-5.18, SE = 1.20, t_9_=-4.33, *p* = 0.0019, R^2^ = 0.68; host tissue C: N: β =-0.26, SE = 0.10, t_9_=-2.45, *p* = 0.037, R^2^ = 0.40, Fig. 3). The C: N of this tissue was also positively related to the abundance of possible fish prey across ponds (β = 0.015, SE = 0.0052, t_9_ = 2.93, *p* = 0.017, R^2^ = 0.49, Fig. 3), while %N was negatively related to the abundance of possible fish prey (β =-0.17, SE = 0.057, t_9_ = 3.00, *p* = 0.015, R^2^ = 0.50, Fig. 3). Pyloric caeca %P was best explained by a positive correlation with pond TP (β = 0.89, SE = 0.25, t_9_ = 3.637, *p* = 0.0054, R^2^ = 0.60, Fig. 3, Online Resource 1, Table 3). Conversely, pyloric caeca C: P and N: P ratios were best explained by negative relationships with pond TP (host tissue C: P: β =-2.48, SE = 0.79, t_9_=-3.16, *p* = 0.012, R^2^ = 0.53, host tissue N: P: β =-3.43, SE = 1.22, t_9_=-2.82, *p* = 0.021, R^2^ = 0.47), and positive relationships with zooplankton abundance (host tissue C: P: β = 0.072, SE = 0.26, t_8_ = 2.82, *p* = 0.023, R^2^ = 0.50; host tissue N: P: β = 0.10, SE = 0.037, t_8_ = 2.77, *p* = 0.024, R^2^ = 0.49, Fig. 3, Online Resource 1, Table 3). Pyloric caeca C: P was also positively associated with water column TC: TP (β = 0.40, SE = 0.13, t_9_ = 3.08, *p* = 0.013, R^2^ = 0.51, Fig. 3, Online Resource 1, Table 3).

### Variation in Crepidostomum spp. abundance across individual hosts

**Fig. 4:**
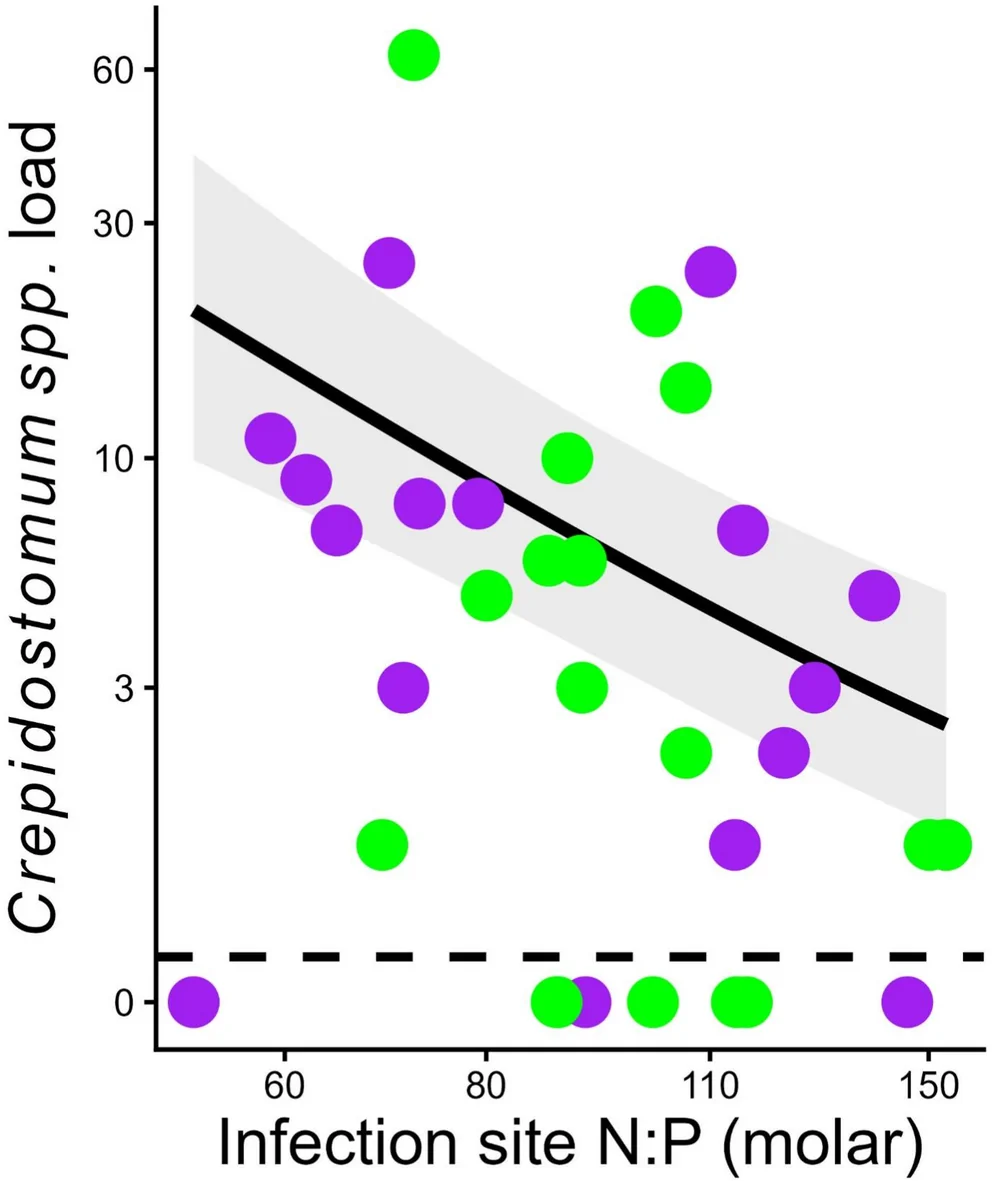
Relationship between the number of *Crepidistomum spp.* in the pyloric caeca of largemouth bass (*N* = 33) and the N:P ratio of that tissue across fertilized (*N* = 5, purple) and unfertilized (*N* = 6, green) ponds. Bass were transferred into ponds with preexisting fish communities and removed after ponds had been fertilized for approximately 3 months. This pattern is based on a truncated poisson that limits the effects of zeros (data below the dotted line) on this trend. Shaded regions indicate standard error

Variation in the abundance of *Crepidostomum spp*. in bass pyloric caeca was best explained by the N: P ratio of this tissue (Online Resource 1, Table 4). Specifically, *Crepidostomum spp*. abundance was inversely related to pyloric caeca N: P ratio (β =-0.56, SE = 0.12, Z = -4.80, *p* < 0.001, mR^2^ = 0.24, cR^2^ = 0.90, Fig. 4).

## Discussion

Understanding how nutrient loading affects the abundance of parasites in freshwater ecosystems is a daunting, but urgent task (McKenzie and Townsend 2007). Previous experiments have elucidated mechanisms driving these effects in lower trophic levels (Bruno et al. 2003; Johnson et al. 2007), but discerning their relevance in natural ecosystems remains difficult. Increasing trophic complexity can further obscure these patterns, especially for upper trophic levels where interactions are less direct and more context dependent. Our experiment in semi-natural experimental ponds shows that ecologically relevant levels of nutrient loading generate variation in basal nutrient availability that are linked, via the stoichiometry of the pyloric caecum, to an increase in the abundance of parasites in this tissue of a predator. Fertilization increased the TP and decreased the C: P and N: P of the water column and FPOM of ponds. This variation in basal nutrient availability was directly related to, and explained as much as 60% of the variation in the %P, C:P and N: P of bass pyloric caeca across ponds (Fig. 3). Ultimately, reduced pyloric caeca N: P ratios were associated with increased *Crepidostomum spp.* abundances in these tissues (Fig. 4). Below, we discuss these trends and other novel insights provided by our assessment of the stoichiometry of an aquatic predator’s pyloric caecum.

Our study contributes to a growing recognition that host stoichiometry, and especially infection site stoichiometry (Stanley et al. 2024), is intricately connected to parasite load (Aalto et al. 2015; Narr and Krist 2015). Our data shows a clear link in the variation of bass pyloric caeca stoichiometry and fertilization-induced shifts in basal nutrients between ponds. As *Crepidostomum spp*. directly ingests the pyloric caecum of the bass (Smyth and Halton 1983), these changes in infection site stoichiometry may improve the balance of nutrients provided to parasites. Experimental evidence on more tractable experimental systems, e.g. *Rhizophydium megarrhizum* fungal parasites in algae (Frenken et al. 2017) and microparasites in *Daphnia* (Narr et al. 2025) suggests that host diet stoichiometry may increase parasite loads when it reduces the difference between host and parasite body stoichiometry. Unfortunately, the small size of this parasite has made it difficult to directly test this possibility (Stanley et al. 2024). We caution against the interpretation of our results that host tissue N: P is consistently negatively related to the abundance of this parasite. While the precise nutritional niche of parasites remain difficult to quantify, they, like all animals, likely have optimal nutrient ratios that facilitate growth and reproduction (Sterner and Elser 2002). If so, the direction of this relationship between parasite abundance and host tissue N: P would shift over the range of tissue nutrient ratios sampled across studies. For example, in a recent analysis comparing parasite abundance to the stoichiometry of multiple infection sites within largemouth bass taken from the Mississippi river, we observed a positive relationship between pyloric caeca N: P and the size of parasitic infracommunities dominated by *Crepidostomum* spp. in this tissue (Stanley et al. 2024). We suspect that the opposing trend in the previous study reflects the lower range of pyloric caeca N: P ratios in that study relative to the present study: in that (2024) study, pyloric caeca N: P ratios ranged from approximately 20 to 100 (Fig. 6, Stanley et al. 2024). In the present study, this range was between approximately 60 and 150. Taken together, these two studies demonstrate the plasticity of the nutrient composition of this host tissue and suggest a potential optimal range of N: P ratios in the pyloric caeca between 60 and 100 for this parasite, with parasite numbers dwindling above or below this range.

It is also likely that host tissue N: P and parasite abundance are linked through a variety pathways, and the dominance of each pathway is context dependent. As a result, different pathways may produce different relationships between host tissue stoichiometry and parasite abundance. The relationship between host tissue stoichiometry and parasite abundance could be mediated by the abundance of the parasite in the host. The parasite loads in this current study are somewhat lower than the parasite loads of the previous study (Stanley et al. 2024). The average abundance of *Crepidostomum* in infected pyloric caeca in this study was 10, and in the previous study it was 30. Two recent studies have shown that individual parasite stoichiometry (which may indicate parasite nutrient requirements) shift as the abundance of parasites increases within a host (Grunberg et al. 2024; Narr et al. 2025) such that larger infrapopulations may have different nutrient requirements than smaller ones. In addition to this potential shift in parasite nutrient requirements, infrapopulation size may also mediate effects of parasites on host tissue. It’s possible that the trends in our previous (Stanley et al. 2024) study were driven by large parasite loads capable of influencing host tissue nutrient content while the trends in our current study were driven by bottom up forces. Detangling these potential feedbacks between parasite and host stoichiometry is likely a critical step in improving the application of stoichiometric theory to host-parasite systems. Another, potentially complimentary mechanism explaining the negative relationship between the N: P of this tissue and its parasite load is that the ingestion of high amounts of an alternative, undetected intermediate host (e.g. burrowing mayflies or crayfish) reduced the N: P ratio of this tissue and simultaneously increased the transfer of parasites to the definitive fish host. If so, nutrient ratios of predator tissues may be more valuable as indicator of transmission processes rather than informing our understanding of the ability of the host to meet the nutritional needs of the parasite. More rigorous methods for sampling intermediate hosts (including the dissection of intermediate hosts to confirm infection) are likely required to isolate the effect of fertilization on transmission of this parasite.

Our study demonstrates that the stoichiometry of nutrient absorbing tissues within upper level consumers can shift in response to the manipulation of basal nutrient availability. While we did not detect clear links between basal resource stoichiometry, intermediate trophic levels, and pyloric caeca stoichiometry, a few lines of evidence from this and another study suggest that fertilization-induced N limitation limited the transfer of N to upper trophic levels. A concomitantly run experiment evaluating the same fertilization regime in a separate group of 14 primarily fishless experimental ponds showed that the reduction in water column TN:TP caused by fertilization was associated with reduced gross primary production and a decline in zooplankton abundance (Sadler et al. 2026). In the present study, we detected a positive relationship between zooplankton abundance and pyloric caeca N: P and C: P ratios (Fig. 3), suggesting that higher abundances of zooplankton facilitated the transfer of N and C (relative to P) to upper trophic levels. Basal N limitation may also explain why fish abundances were positively associated with water column TN concentrations and negatively associated with water column TC:TN ratios (Fig. 2). We note that, unlike zooplankton (which appear to have increased the transfer of N to pyloric caeca), higher fish abundances were associated with lower pyloric caeca %N and positive C:N ratios (Fig. 3). The positive relationship between planktivorous fish abundance and pyloric caeca C: N ratios is consistent with other work showing that pyloric caeca C: N ratios increase with fish condition (Stanley et al. 2024). Taken together, the relationships we have documented between basal resources and pyloric caeca nutrient ratios suggest that high concentrations of nonlimiting nutrients (here P) can be detected in the digestive tissue of a predator. This may not be surprising given that, at high dietary concentrations, passive paracellular diffusion dominates P uptake in the pyloric caeca, and, unlike active P absorption processes, passive diffusion can allow for the absorption of large amounts of nutrients (Sugiura 2024).

It is not particularly surprising that we were unable to detect clear effects of fertilization on the intermediate trophic levels between basal nutrients and bass. We conducted our experiment in complex, semi-natural ponds with prexisiting planktivorous fish communities and piscivorous bass. Consistent with an improved ability to detect responses to nutrient availability in simpler ecosystems, Sadler et al. (2026) were only able to detect relationships between basal nutrients and zooplankton when (four) ponds with fish were excluded from their (fourteen pond) dataset. The time required to sample each component of these complex communities constrained the number of ponds in our study and limited our ability to evaluate the strength of hypothetical pathways using more sophisticated statistical analyses such as structural equation modeling. Although this approach was not feasible with our sample size, it provides a useful framework for evaluating direct and indirect relationships among multiple hosts and nutrient pools in complex ecological systems. Future studies designed with larger sample sizes and structural equation modeling in mind could offer stronger tests of hypothesized nutrient pathways and improve our understanding of nutrient movement through multi-host parasite systems. We also used semi-quantitative, standardized sampling methods to sample these intermediate groups (e.g. timed dip netting over a set distance) that were unlikely to detect all invertebrate groups equally well (Narr and Krist 2019), and we only sampled twice (once before and once during fertilization) over the duration of the experiment. Rigorous estimates of the flow of nutrients through even a single invertebrate group (i.e. secondary production) can be logistically challenging. The relationship we observed between nutrient ratios at the base of the food web and those in the pyloric caeca of a predator suggests that the digestive tissues of predators may amalgamate the response of lower trophic levels to nutrient loading. If so, comparing the nutrient ratios of these tissues across ecosystems could improve our understanding of bottom-up effects on predator nutrition and provide an early indication of nutrient loads.

Ponds are important for aquaculture, especially private aquaculture (Boyd and Tucker 2012). As natural fisheries stocks become further depleted, ponds managed for fish will become increasingly important (Egna and Boyd 1997). Fertilization is often used to increase production in these ponds because it is relatively cheap and low maintenance compared to buying pellet-based food for fish. We based the amount and frequency of fertilization in this experiment on recommendations for new ponds from the Illinois Department of Natural Resources (2017) in literature intended for private landowner use. The fertilizer we used (liquid ammonium polyphosphate) is easily obtainable, water soluble, and widely used for fertilization of ponds and plants (like turf grass). This treatment is thus comparable to what private landowners might use to boost productivity in their ponds. The conditions created by this treatment might also be similar to those of ponds on golf courses or on other manicured lawns, as per the manufacturers, Plant Food Inc, recommendations (2017). We did not observe a direct effect of this fertilization regime on bass length or *Crepidostomum spp.* abundance. However, the addition of fertilizer increased water column TP levels which explained 47% of the variation in pyloric caeca N: P across ponds. Ultimately, these reduced tissue N: P ratios were associated with higher loads of our focal parasite. Our findings add to a growing body of work showing indirect links between nutrient enrichment and parasite loads, extending these trends into more complex systems. More research is still needed to understand the mechanisms behind these shifts as nutrient enrichment both intentionally and unintentionally is expected to continue. 

## Supplementary Information

Below is the link to the electronic supplementary material.

Supplementary Material 1

## Data Availability

The datasets used and/or analysed during the current study are available from the corresponding author on reasonable request.

## Code Availability

The code used in the current study is available from the corresponding author on reasonable request.
