# Supplementary material for: Host tissue stoichiometry links nutrient availability to parasite abundance in a piscivorous fish
Source: Oecologia. 2026 Sep 4;208(10):125. doi: 10.1007/s00442-026-05971-w (PMC13545112; doi:10.1007/s00442-026-05971-w)
Supplement: Supplementary file 1 [file 442_2026_5971_MOESM1_ESM.docx]

**Online Resource 1**

Host tissue stoichiometry links nutrient availability to parasite abundance in a piscivorous fish

Adrienne Stanley^1^, Isabella Sadler^1^, Scott Binger^1^, Charlotte F Narr ^1^*

^1^School of Biological Sciences, Southern Illinois University in Carbondale, Carbondale, IL, USA

^[*](mailto:*charlotte.narr@siu.edu)^[charlotte.narr@siu.edu](mailto:*charlotte.narr@siu.edu), 1 618-453-4109

**Fig 1** Total water column mean and minimum dissolved oxygen (DO), carbon (TC), nitrogen (TN), phosphorus (TP), and molar ratios of these nutrients (TC:TN, TC:TP, and TN:TP) in 6 fertilized (purple) and 6 unfertilized (green) ponds between June 24^th^ (day of year 175) and September 21^st^ (day of year 264) in 2021. The dotted vertical line shows the day fertilization was initiated in fertilized ponds (July 9^th^, day of year 190). Each line represents the value from a single pond.

Table 1. The number and type of fish caught seining twelve 0.04-hectare ponds located at the outdoor experimental pond facility at the Touch of Nature Environmental Center outside of Carbondale, IL. Ponds were seined from June 22^nd^ through July 2^nd^, 2021 using a 6 foot deep seine with 1/8 inch mesh.

| Pond | Crappie | Bluegill | Geen Sunfish | Largemouth Bass | Golden Shiner |
| --- | --- | --- | --- | --- | --- |
| 38 | 589 |  |  |  | 23 |
| 39 | 101 |  |  |  | 2 |
| 40 |  |  |  |  |  |
| 44 |  |  | 3659 |  |  |
| 45 |  |  |  |  |  |
| 47 |  |  |  | 319 |  |
| 48 | 470 |  |  |  |  |
| 49 | 411 |  |  |  |  |
| 55 |  |  |  | 356 |  |
| 71 | 359 |  |  |  |  |
| 72 | 90 | 330 |  |  |  |
| 88 | 79 | 1064 |  |  |  |

Table 2: Model selection for linear models predicting natural log-tranformed (ln) invertebrate abundances and rank transformed fish and clam abundances (individuals/pond) in 5 fertilized and 6 unfertilized experimental ponds based on the reciprocal variable, water column dissolved oxygen (DO, mean and minimum, min), total carbon (TC), nitrogen (TN), phosphorus (TP), and natural log-transformed molar ratios of these nutrients (ln TC:TN, ln TC:TP, and ln TN:TP), and fine particulate organic matter nutrient concentration (µgL^-1^ of C, N, P) and nutrient ratios (C:N, C:P, and N:P). Number of parameters (K), change in AIC_C_ compared to the best-ranked model (ΔAIC_C_), Akaike model weights (*W*), and log likelihood estimate (*LL*) for top models (ΔAIC_C_ < 2) are shown in bold.

| Response | Predictor | K | ΔAIC_C_ | *W* | *LL* | R2 |
| --- | --- | --- | --- | --- | --- | --- |
| ln Benthic Invertebrate abundance | **ln Pre Benthic Inverts** | **3** | **0.00** | **0.39** | **-9.24** | **0.50** |
|  | ln FPOM N | 3 | 3.10 | 0.08 | -10.79 | 0.33 |
|  | ln TN:TP | 3 | 3.23 | 0.08 | -10.86 | 0.32 |
|  | ln FPOM N:P | 3 | 3.38 | 0.07 | -10.93 | 0.32 |
|  | Fertilization | 3 | 3.48 | 0.07 | -10.98 | 0.31 |
|  | Null | 2 | 3.61 | 0.06 | -13.01 | 0.00 |
|  | ln TC:TP | 3 | 4.24 | 0.05 | -11.36 | 0.26 |
|  | ln FPOM C | 3 | 5.16 | 0.03 | -11.82 | 0.19 |
|  | TP | 3 | 5.77 | 0.02 | -12.12 | 0.15 |
|  | TN | 3 | 5.93 | 0.02 | -12.21 | 0.14 |
|  | ln FPOM C:P | 3 | 5.95 | 0.02 | -12.21 | 0.14 |
|  | ln FPOM C:N | 3 | 6.11 | 0.02 | -12.30 | 0.12 |
|  | rank Fish | 3 | 6.12 | 0.02 | -12.30 | 0.12 |
|  | Mean DO | 3 | 6.54 | 0.01 | -12.51 | 0.09 |
|  | ln TC:TN | 3 | 6.57 | 0.01 | -12.53 | 0.08 |
|  | ln FPOM P | 3 | 6.78 | 0.01 | -12.63 | 0.07 |
|  | Min DO | 3 | 6.93 | 0.01 | -12.71 | 0.05 |
|  | TC | 3 | 7.54 | 0.01 | -13.01 | 0.00 |
| ln Zooplankton abundance | **Null** | **2** | **0.00** | **0.27** | **-15.32** | **0.00** |
|  | rank Fish | 3 | 2.40 | 0.08 | -14.37 | 0.17 |
|  | ln FPOM P | 3 | 2.78 | 0.07 | -14.56 | 0.14 |
|  | ln TC:TN | 3 | 2.92 | 0.06 | -14.64 | 0.13 |
|  | Fertilization | 3 | 3.19 | 0.05 | -14.77 | 0.10 |
|  | TN | 3 | 3.45 | 0.05 | -14.90 | 0.08 |
|  | TP | 3 | 3.73 | 0.04 | -15.04 | 0.05 |
|  | ln Pre Zooplankton | 3 | 3.78 | 0.04 | -15.07 | 0.05 |
|  | ln TC:TP | 3 | 3.79 | 0.04 | -15.07 | 0.05 |
|  | ln FPOM N | 3 | 3.80 | 0.04 | -15.07 | 0.05 |
|  | ln FPOM C | 3 | 3.97 | 0.04 | -15.16 | 0.03 |
|  | ln FPOM C:N | 3 | 4.18 | 0.03 | -15.27 | 0.01 |
|  | Mean DO | 3 | 4.21 | 0.03 | -15.28 | 0.01 |
|  | ln TN:TP | 3 | 4.24 | 0.03 | -15.30 | 0.00 |
|  | Min DO | 3 | 4.25 | 0.03 | -15.30 | 0.00 |
|  | ln FPOM C:P | 3 | 4.27 | 0.03 | -15.31 | 0.00 |
|  | ln FPOM N:P | 3 | 4.28 | 0.03 | -15.32 | 0.00 |
|  | TC | 3 | 4.29 | 0.03 | -15.32 | 0.00 |
| Rank Fish prey abundance | **ln TC:TN** | **3** | **0.00** | **0.43** | **-24.68** | **0.48** |
|  | **TN** | **3** | **1.96** | **0.16** | **-25.66** | **0.38** |
|  | Null | 2 | 3.26 | 0.08 | -28.27 | 0.00 |
|  | ln Zooplankton | 3 | 4.30 | 0.05 | -26.83 | 0.23 |
|  | ln TN:TP | 3 | 5.24 | 0.03 | -27.30 | 0.16 |
|  | rank Pre Fish | 3 | 5.27 | 0.03 | -27.31 | 0.16 |
|  | ln FPOM N | 3 | 5.51 | 0.03 | -27.43 | 0.14 |
|  | ln FPOM N:P | 3 | 5.64 | 0.03 | -27.50 | 0.13 |
|  | ln Benthic inverts | 3 | 5.77 | 0.02 | -27.56 | 0.12 |
|  | ln FPOM C | 3 | 6.18 | 0.02 | -27.77 | 0.09 |
|  | Mean DO | 3 | 6.45 | 0.02 | -27.90 | 0.07 |
|  | ln FPOM C:P | 3 | 6.57 | 0.02 | -27.96 | 0.06 |
|  | ln FPOM P | 3 | 6.75 | 0.01 | -28.05 | 0.04 |
|  | ln FPOM C:N | 3 | 6.76 | 0.01 | -28.06 | 0.04 |
|  | TP | 3 | 7.02 | 0.01 | -28.19 | 0.02 |
|  | Min DO | 3 | 7.04 | 0.01 | -28.20 | 0.01 |
|  | Fertilization | 3 | 7.04 | 0.01 | -28.20 | 0.01 |
|  | ln TC:TP | 3 | 7.16 | 0.01 | -28.26 | 0.00 |
|  | TC | 3 | 7.19 | 0.01 | -28.27 | 0.00 |
| Bass Length | **ln FPOM N:P** | **3** | **0.00** | **0.14** | **-48.29** | **0.34** |
|  | **ln FPOM N** | **3** | **0.37** | **0.12** | **-48.48** | **0.31** |
|  | **Null** | **2** | **0.58** | **0.10** | **-50.55** | **0.00** |
|  | TN | 3 | 1.27 | 0.07 | -48.93 | 0.26 |
|  | ln FPOM C | 3 | 1.46 | 0.07 | -49.02 | 0.24 |
|  | Fertilization | 3 | 1.50 | 0.07 | -49.04 | 0.24 |
|  | ln Zooplankton | 3 | 1.56 | 0.06 | -49.07 | 0.24 |
|  | ln Benthic inverts | 3 | 1.57 | 0.06 | -49.08 | 0.23 |
|  | ln FPOM C:P | 3 | 1.90 | 0.05 | -49.25 | 0.21 |
|  | ln TN:TP | 3 | 1.98 | 0.05 | -49.29 | 0.21 |
|  | ln TC:TN | 3 | 2.53 | 0.04 | -49.56 | 0.17 |
|  | rank Fish | 3 | 2.58 | 0.04 | -49.58 | 0.16 |
|  | ln TC:TP | 3 | 3.72 | 0.02 | -50.16 | 0.07 |
|  | ln FPOM P | 3 | 3.98 | 0.02 | -50.28 | 0.05 |
|  | TC | 3 | 4.04 | 0.02 | -50.31 | 0.04 |
|  | ln FPOM C:N | 3 | 4.34 | 0.02 | -50.46 | 0.02 |
|  | Min DO | 3 | 4.40 | 0.02 | -50.49 | 0.01 |
|  | TP | 3 | 4.41 | 0.02 | -50.50 | 0.01 |
|  | Mean DO | 3 | 4.49 | 0.01 | -50.54 | 0.00 |
| Rank Clam Abundance | Null | 2 | 0.00 | 0.22 | -26.62 | 0.00 |
|  | TN | 3 | 1.41 | 0.11 | -25.36 | 0.21 |
|  | ln TC:TN | 3 | 2.26 | 0.07 | -25.79 | 0.14 |
|  | rank Fish | 3 | 2.31 | 0.07 | -25.82 | 0.14 |
|  | Pre Clam | 3 | 2.69 | 0.06 | -26.00 | 0.11 |
|  | TP | 3 | 3.19 | 0.05 | -26.26 | 0.07 |
|  | ln FPOM C:N | 3 | 3.42 | 0.04 | -26.37 | 0.05 |
|  | Fertilization | 3 | 3.47 | 0.04 | -26.40 | 0.04 |
|  | ln FPOM N:P | 3 | 3.59 | 0.04 | -26.45 | 0.03 |
|  | ln FPOM N | 3 | 3.66 | 0.04 | -26.49 | 0.03 |
|  | ln TC:TN | 3 | 3.68 | 0.04 | -26.50 | 0.02 |
|  | ln TN:TP | 3 | 3.71 | 0.03 | -26.51 | 0.02 |
|  | ln TC:TP | 3 | 3.74 | 0.03 | -26.53 | 0.02 |
|  | Mean DO | 3 | 3.82 | 0.03 | -26.57 | 0.01 |
|  | ln FPOM C | 3 | 3.85 | 0.03 | -26.58 | 0.01 |
|  | ln FPOM C:P | 3 | 3.88 | 0.03 | -26.60 | 0.01 |
|  | Min DO | 3 | 3.89 | 0.03 | -26.60 | 0.00 |
|  | ln FPOM P | 3 | 3.89 | 0.03 | -26.61 | 0.00 |

Table 3: Model selection for linear models predicting average pyloric caeca % carbon (C), nitrogen (N), and phosphorus (P) and natural log-transformed (ln) molar C:N, C:P, and N:P ratios in 5 fertilized and 6 unfertilized experimental ponds based on natural log-transformed invertebrate abundances and zooplankton abundances, rank transformed possible fish prey abundances (individuals/pond), water column dissolved oxygen (DO, mean and minimum, min), total carbon (TC), nitrogen (TN), phosphorus (TP), natural log-transformed molar ratios of these nutrients (ln TC:TN, ln TC:TP, and ln TN:TP), and fine particulate organic matter nutrient concentration (µgL^-1^ of C, N, P) and nutrient ratios (C:N, C:P, and N:P). Number of parameters (K), change in AIC_C_ compared to the best-ranked model (ΔAIC_C_), Akaike model weights (*W*), and log likelihood estimate (*LL*) for top models (ΔAIC_C_ < 2) are shown in bold.

| **Nutrient** | **Predictors** | **K** | **ΔAIC_C_** | ***W*** | ***LL*** | **R^2^** |
| --- | --- | --- | --- | --- | --- | --- |
| PC %C | **ln FPOM C:N** | **3** | **0.00** | **0.90** | **-10.18** | **0.68** |
|  | ln Zooplankton | 3 | 6.85 | 0.03 | -13.32 | 0.07 |
|  | Rank Fish | 3 | 8.11 | 0.02 | -14.24 | 0.32 |
|  | Null | 2 | 8.45 | 0.01 | -16.37 | 0.00 |
|  | ln FPOM C:P | 3 | 10.05 | 0.01 | -15.20 | 0.19 |
|  | TN | 3 | 10.50 | 0.00 | -15.43 | 0.16 |
|  | TP | 3 | 11.12 | 0.00 | -15.74 | 0.11 |
|  | ln FPOM C | 3 | 11.23 | 0.00 | -15.79 | 0.10 |
|  | TP | 3 | 11.42 | 0.00 | -15.89 | 0.08 |
|  | ln Benthic Inverts | 3 | 11.95 | 0.00 | -16.15 | 0.04 |
|  | ln FPOM N:P | 3 | 12.11 | 0.00 | -16.23 | 0.02 |
|  | TC:TN | 3 | 12.12 | 0.00 | -16.24 | 0.02 |
|  | Fertilization | 3 | 12.13 | 0.00 | -16.25 | 0.02 |
|  | Min DO | 3 | 12.20 | 0.00 | -16.28 | 0.02 |
|  | ln FPOM P | 3 | 12.29 | 0.00 | -16.32 | 0.01 |
|  | TN:TP | 3 | 12.29 | 0.00 | -16.32 | 0.01 |
|  | Mean DO | 3 | 12.30 | 0.00 | -16.33 | 0.01 |
|  | ln FPOM N | 3 | 12.31 | 0.00 | -16.34 | 0.01 |
|  | Length | 3 | 12.37 | 0.00 | -16.36 | 0.00 |
|  | TC:TP | 3 | 12.37 | 0.00 | -16.36 | 0.00 |
| PC %N | **Rank Fish** | **3** | **0.00** | **0.38** | **-8.85** | **0.50** |
|  | **ln Zooplankton** | **3** | **1.69** | **0.16** | **-9.41** | **0.27** |
|  | TN | 3 | 2.36 | 0.12 | -10.03 | 0.38 |
|  | Null | 2 | 3.70 | 0.06 | -12.67 | 0.00 |
|  | ln FPOM C:N | 3 | 4.09 | 0.05 | -10.90 | 0.28 |
|  | TP | 3 | 4.13 | 0.05 | -10.92 | 0.27 |
|  | TP | 3 | 4.58 | 0.04 | -11.14 | 0.24 |
|  | ln FPOM P | 3 | 4.97 | 0.03 | -11.34 | 0.22 |
|  | Fertilization | 3 | 6.16 | 0.02 | -11.93 | 0.13 |
|  | ln FPOM C:P | 3 | 6.68 | 0.01 | -12.19 | 0.08 |
|  | TC:TN | 3 | 6.75 | 0.01 | -12.23 | 0.08 |
|  | ln FPOM N | 3 | 7.30 | 0.01 | -12.50 | 0.03 |
|  | TC:TP | 3 | 7.31 | 0.01 | -12.51 | 0.03 |
|  | Min DO | 3 | 7.48 | 0.01 | -12.59 | 0.01 |
|  | ln FPOM N:P | 3 | 7.52 | 0.01 | -12.61 | 0.01 |
|  | Length | 3 | 7.57 | 0.01 | -12.64 | 0.01 |
|  | TN:TP | 3 | 7.59 | 0.01 | -12.65 | 0.00 |
|  | ln Benthic Inverts | 3 | 7.62 | 0.01 | -12.66 | 0.00 |
|  | ln FPOM C | 3 | 7.63 | 0.01 | -12.67 | 0.00 |
|  | Mean DO | 3 | 7.63 | 0.01 | -12.67 | 0.00 |
| PC %P | **TP** | **3** | **0.00** | **0.58** | **24.09** | **0.60** |
|  | TC:TP | 3 | 2.64 | 0.16 | 22.77 | 0.49 |
|  | Fertilization | 3 | 4.52 | 0.06 | 21.83 | 0.39 |
|  | ln FPOM C:P | 3 | 5.81 | 0.03 | 21.19 | 0.31 |
|  | Null | 2 | 6.02 | 0.03 | 19.12 | 0.00 |
|  | ln FPOM N:P | 3 | 6.74 | 0.02 | 20.72 | 0.25 |
|  | TN:TP | 3 | 7.11 | 0.02 | 20.54 | 0.23 |
|  | ln Zooplankton | 3 | 7.15 | 0.02 | 20.80 | 0.38 |
|  | ln FPOM C | 3 | 7.41 | 0.01 | 20.39 | 0.21 |
|  | ln FPOM N | 3 | 8.16 | 0.01 | 20.01 | 0.15 |
|  | Rank Fish | 3 | 8.16 | 0.01 | 20.01 | 0.15 |
|  | ln Benthic Inverts | 3 | 8.42 | 0.01 | 19.88 | 0.13 |
|  | Mean DO | 3 | 8.63 | 0.01 | 19.78 | 0.11 |
|  | ln FPOM C:N | 3 | 8.93 | 0.01 | 19.63 | 0.09 |
|  | Min DO | 3 | 8.96 | 0.01 | 19.61 | 0.09 |
|  | Length | 3 | 9.31 | 0.01 | 19.43 | 0.06 |
|  | TP | 3 | 9.35 | 0.01 | 19.42 | 0.05 |
|  | TN | 3 | 9.65 | 0.00 | 19.27 | 0.03 |
|  | TC:TN | 3 | 9.89 | 0.00 | 19.15 | 0.01 |
|  | ln FPOM P | 3 | 9.94 | 0.00 | 19.12 | 0.00 |
| ln PC C:N | **Rank Fish** | **3** | **0.00** | **0.40** | **17.51** | **0.49** |
|  | **ln FPOM C:N** | **3** | **1.74** | **0.17** | **16.65** | **0.40** |
|  | TN | 3 | 2.73 | 0.10 | 16.15 | 0.34 |
|  | Null | 2 | 3.44 | 0.07 | 13.83 | 0.00 |
|  | TP | 3 | 4.34 | 0.05 | 15.34 | 0.24 |
|  | TP | 3 | 4.85 | 0.04 | 15.09 | 0.20 |
|  | ln FPOM P | 3 | 5.72 | 0.02 | 14.65 | 0.14 |
|  | ln FPOM C:P | 3 | 6.04 | 0.02 | 14.49 | 0.11 |
|  | Fertilization | 3 | 6.33 | 0.02 | 14.35 | 0.09 |
|  | TC:TN | 3 | 6.60 | 0.01 | 14.21 | 0.07 |
|  | ln Zooplankton | 3 | 6.63 | 0.01 | 14.48 | 0.21 |
|  | TC:TP | 3 | 7.17 | 0.01 | 13.93 | 0.02 |
|  | ln FPOM N | 3 | 7.23 | 0.01 | 13.90 | 0.01 |
|  | ln FPOM N:P | 3 | 7.23 | 0.01 | 13.90 | 0.01 |
|  | ln Benthic Inverts | 3 | 7.29 | 0.01 | 13.87 | 0.01 |
|  | ln FPOM C | 3 | 7.29 | 0.01 | 13.87 | 0.01 |
|  | TN:TP | 3 | 7.31 | 0.01 | 13.86 | 0.01 |
|  | Length | 3 | 7.32 | 0.01 | 13.86 | 0.00 |
|  | Min DO | 3 | 7.33 | 0.01 | 13.85 | 0.00 |
|  | Mean DO | 3 | 7.36 | 0.01 | 13.83 | 0.00 |
| ln PC C:P | **TP** | **3** | **0.00** | **0.38** | **11.91** | **0.59** |
|  | **TC:TP** | **3** | **0.52** | **0.29** | **11.65** | **0.56** |
|  | **ln Zooplankton** | **3** | **1.92** | **0.15** | **10.95** | **0.50** |
|  | Null | 2 | 4.53 | 0.04 | 7.50 | 0.00 |
|  | Fertilization | 3 | 5.35 | 0.03 | 9.23 | 0.29 |
|  | Mean DO | 3 | 6.92 | 0.01 | 8.45 | 0.17 |
|  | TN:TP | 3 | 6.99 | 0.01 | 8.42 | 0.17 |
|  | Min DO | 3 | 7.07 | 0.01 | 8.38 | 0.16 |
|  | ln FPOM C:P | 3 | 7.33 | 0.01 | 8.24 | 0.14 |
|  | ln FPOM N:P | 3 | 7.38 | 0.01 | 8.22 | 0.13 |
|  | Rank Fish | 3 | 7.55 | 0.01 | 8.14 | 0.12 |
|  | ln Benthic Inverts | 3 | 7.63 | 0.01 | 8.10 | 0.11 |
|  | TN | 3 | 8.04 | 0.01 | 7.89 | 0.07 |
|  | TC:TN | 3 | 8.17 | 0.01 | 7.82 | 0.06 |
|  | ln FPOM C | 3 | 8.20 | 0.01 | 7.81 | 0.06 |
|  | ln FPOM N | 3 | 8.29 | 0.01 | 7.76 | 0.05 |
|  | TP | 3 | 8.51 | 0.01 | 7.66 | 0.03 |
|  | ln FPOM P | 3 | 8.65 | 0.01 | 7.58 | 0.02 |
|  | ln FPOM C:N | 3 | 8.72 | 0.00 | 7.55 | 0.01 |
|  | Length | 3 | 8.81 | 0.00 | 7.50 | 0.00 |
| ln PC N:P | **TP** | **3** | **0.00** | **0.38** | **7.91** | **0.54** |
|  | **ln Zooplankton** | **3** | **1.05** | **0.22** | **7.38** | **0.49** |
|  | Null | 2 | 3.48 | 0.07 | 4.03 | 0.00 |
|  | Rank Fish | 3 | 3.79 | 0.06 | 6.01 | 0.33 |
|  | TN | 3 | 4.02 | 0.05 | 5.90 | 0.31 |
|  | TC:TP | 3 | 4.34 | 0.04 | 5.74 | 0.29 |
|  | Fertilization | 3 | 5.64 | 0.02 | 5.09 | 0.19 |
|  | TP | 3 | 5.93 | 0.02 | 4.94 | 0.17 |
|  | ln FPOM C:N | 3 | 5.99 | 0.02 | 4.91 | 0.16 |
|  | ln FPOM C:P | 3 | 6.32 | 0.02 | 4.75 | 0.13 |
|  | TC:TN | 3 | 6.47 | 0.01 | 4.67 | 0.12 |
|  | ln FPOM P | 3 | 6.49 | 0.01 | 4.66 | 0.12 |
|  | Min DO | 3 | 6.64 | 0.01 | 4.59 | 0.11 |
|  | Mean DO | 3 | 6.67 | 0.01 | 4.57 | 0.10 |
|  | ln FPOM N:P | 3 | 7.22 | 0.01 | 4.30 | 0.05 |
|  | Length | 3 | 7.28 | 0.01 | 4.27 | 0.05 |
|  | TN:TP | 3 | 7.43 | 0.01 | 4.19 | 0.03 |
|  | ln FPOM C | 3 | 7.62 | 0.01 | 4.10 | 0.01 |
|  | ln Benthic Inverts | 3 | 7.64 | 0.01 | 4.09 | 0.01 |
|  | ln FPOM N | 3 | 7.76 | 0.01 | 4.03 | 0.00 |

Table 4: Results of model selection for truncated poisson mixed models predicting the number of *Crepidistomum spp*. in the pyloric caeca of largemouth bass based on a random effect of pond and bass pyloric caeca (PC) nutrient content (%C, N, P) or nutrient ratios (C:N, C:P, and N:P), pond fertilization status, the natural log-transformed (ln) average abundance of sphaerid clams (clam) over the course of the experiment, ln invertebrate abundances (individuals/L, invert), rank transformed possible fish prey abundances (individuals/pond), water column dissolved oxygen (mean and minimum, minDO), total carbon (TC), nitrogen (TN), phosphorus (TP), and natural log-transformed molar ratios of these nutrients (ln TC:TN, ln TC:TP, and ln TN:TP), fine particulate organic matter nutrient concentration (µgL^-1^ of C, N, P) and nutrient ratios (C:N, C:P, and N:P), and a null model. Number of parameters (K), change in AIC_C_ compared to the best-ranked model (ΔAIC_C_), Akaike model weights (*W*), log likelihood estimate (*LL*), and both marginal (Marg R^2^) and conditional R-squared (Cond R^2^) for top models (ΔAIC_C_ < 2) are shown in bold.

| Model Predictors | K | ΔAIC_C_ | *W* | *LL* | Marg R^2^ | Cond R^2^ |
| --- | --- | --- | --- | --- | --- | --- |
| **PC N:P** | **5** | **0.00** | **0.26** | **-104.47** | **0.24** | **0.90** |
| **PC N:P +ln Fish** | **6** | **1.79** | **0.11** | **-103.86** | **0.29** | **0.88** |
| PC N:P + ln Benthic Inverts | 6 | 2.12 | 0.09 | -104.03 | 0.37 | 0.91 |
| PC N:P + ln FPOM P | 6 | 2.44 | 0.08 | -104.19 | 0.29 | 0.89 |
| PC N:P + Clam | 6 | 2.60 | 0.07 | -104.27 | 0.25 | 0.89 |
| PC N:P + ln min DO | 6 | 2.70 | 0.07 | -104.32 | 0.26 | 0.89 |
| PC N:P + ln FPOM N | 6 | 2.96 | 0.06 | -104.45 | 0.25 | 0.89 |
| PC N:P + ln FPOM C | 6 | 2.99 | 0.06 | -104.46 | 0.24 | 0.89 |
| PC N:P + ln mean DO | 6 | 3.01 | 0.06 | -104.47 | 0.25 | 0.90 |
| PC N:P + ln Zooplankton | 6 | 3.01 | 0.06 | -104.47 | 0.25 | 0.90 |
| PC %P | 5 | 4.88 | 0.02 | -106.91 | 0.17 | 0.87 |
| PC %P + ln Fish | 6 | 7.11 | 0.01 | -106.52 | 0.20 | 0.87 |
| PC C:P | 5 | 7.26 | 0.01 | -108.10 | 0.17 | 0.87 |
| PC %P + FPOM P | 6 | 7.44 | 0.01 | -106.69 | 0.22 | 0.87 |
| PC %P + ln min DO | 6 | 7.45 | 0.01 | -106.69 | 0.19 | 0.87 |
| PC %P + ln Benthic Invert | 6 | 7.47 | 0.01 | -106.70 | 0.27 | 0.88 |
| PC %P + ln Clam | 6 | 7.66 | 0.01 | -106.80 | 0.18 | 0.87 |
| PC %P + ln Zooplankton | 6 | 7.75 | 0.01 | -106.84 | 0.19 | 0.88 |
| PC %P + ln FPOM N | 6 | 7.85 | 0.01 | -106.89 | 0.18 | 0.87 |
| PC %P + ln FPOM C | 6 | 7.89 | 0.01 | -106.91 | 0.17 | 0.87 |
